# Application of machine learning methods to histone methylation ChIP-Seq data reveals H4R3me2 globally represses gene expression

**DOI:** 10.1186/1471-2105-11-396

**Published:** 2010-07-23

**Authors:** Xiaojiang Xu, Stephen Hoang, Marty W Mayo, Stefan Bekiranov

**Affiliations:** 1 Department of Biochemistry and Molecular Genetics, University of Virginia Health System, Charlottesville, Virginia, USA

## Abstract

**Background:**

In the last decade, biochemical studies have revealed that epigenetic modifications including histone modifications, histone variants and DNA methylation form a complex network that regulate the state of chromatin and processes that depend on it including transcription and DNA replication. Currently, a large number of these epigenetic modifications are being mapped in a variety of cell lines at different stages of development using high throughput sequencing by members of the ENCODE consortium, the NIH Roadmap Epigenomics Program and the Human Epigenome Project. An extremely promising and underexplored area of research is the application of machine learning methods, which are designed to construct predictive network models, to these large-scale epigenomic data sets.

**Results:**

Using a ChIP-Seq data set of 20 histone lysine and arginine methylations and histone variant H2A.Z in human CD4^+ ^T-cells, we built predictive models of gene expression as a function of histone modification/variant levels using Multilinear (ML) Regression and Multivariate Adaptive Regression Splines (MARS). Along with extensive crosstalk among the 20 histone methylations, we found H4R3me2 was the most and second most globally repressive histone methylation among the 20 studied in the ML and MARS models, respectively. In support of our finding, a number of experimental studies show that PRMT5-catalyzed symmetric dimethylation of H4R3 is associated with repression of gene expression. This includes a recent study, which demonstrated that H4R3me2 is required for DNMT3A-mediated DNA methylation--a known global repressor of gene expression.

**Conclusion:**

In stark contrast to univariate analysis of the relationship between H4R3me2 and gene expression levels, our study showed that the regulatory role of some modifications like H4R3me2 is masked by confounding variables, but can be elucidated by multivariate/systems-level approaches.

## Background

Histones are subjected to numerous modifications, including methylation, acetylation and phosphorylation. Over 60 different amino acid residues of the histones, detected by specific antibodies or mass spectrometry, can be modified. They regulate a number of important processes on DNA, including transcription [1,2]. Extensive studies comparing histone modification and transcription levels have established that histone methylation is associated with either transcriptional repression or activation. A number of marks have been classified as "activating" transcription including H3 lysine 4 tri-methyl (H3K4me3) and H3 lysine 36 tri-methyl (H3K36me3) and "repressing" transcription including H3 lysine 27 tri-methyl (H3K27me3) [1,2]. These modifications can be recognized by chromatin remodeling proteins (readers), which render chromatin in either "open", transcriptionally permissive conformations or "closed", DNA-inaccessible conformations, respectively [1,2].

A simple question that emerges is: Why does the cell require ~100 or more modifications to maintain two (i.e., open and closed) or a handful of chromatin states? The histone code hypothesis was developed to address this question. The histone code hypothesis "suggested that distinct functional consequences result from histone modifications and that a given outcome is encoded in the precise nature and pattern of marks" [3-6]. A challenge to the hypothesis has been the identification of multiple readers for a single modification, thereby confounding "a simple one-mark-to-one-module type of decoding" [3]. A framework that keeps the histone code hypothesis intact and addresses this criticism is the phenomenon of multivalency--the cooperative engagement of several linked substrates by a species with more than one discrete interacting surface [2,3]. In other words, chromatin regulatory proteins and their associated complexes write, read and erase multiple histone modifications simultaneously. It has been suggested that multivalency may be widespread in chromatin regulation. Indeed, a number of recent studies are uncovering patterns of coexisting histone marks, extensive crosstalk among different modifications as well as multiple effector proteins on the same complex [2,3,7-9].

Using ChIP-chip and ChIP-Seq, bivalent domains of H3K4me3 and H3K27me3 were observed at genes encoding developmentally important transcription factors in embryonic stem cells [10-12]. It is suggested that these genes are transcriptionally silent but poised for activation during development. Indeed, in differentiated cells the vast majority of bivalent domains (93/97) resolved into either K4me3 (active genes) or K27me3 (repressed genes). Consistent with the idea of widespread multivalency, it is notable that two "opposing" marks were assayed on a genomic scale and were found to occur in bivalent domains. It raises the question: If many more marks were mapped, would we find widespread multivalencies?

To help address these questions we applied two machine learning methods, Stepwise Multilinear Regression and Multivariate Adaptive Regression Splines (MARS) [13], to genome-wide ChIP-Seq maps of 20 histone lysine and arginine methylations and histone variant H2A.Z in CD4^+ ^T-cells [14]. We hypothesize that inclusion of two (bivalent) and three (trivalent) interacting cross-terms in the model can reveal (1) putative cross-regulation or multivalent relationships between histone modifications and (2) a global view of the epigenetic regulatory network. Specifically, we first estimate the enrichment level of each modification using a new, model-based approach, which accounts for the characteristic spatial distribution of each modification across genes. With the enrichment levels as inputs and normalized log_2 _gene expression levels as output, we build the multilinear (ML) model from a set of 21 single or monovalent inputs, 210 bivalent inputs and 1330 trivalent inputs. For the MARS model, the 21 monovalent amplitudes were supplied as input and the bi- and trivalent interacting terms were added as part of the model optimization procedure. Using 10-fold cross validation and requiring terms to appear in 5 of 10 training models, our best ML model contained 7, 8 and 8 mono-, bi-and trivalent terms, respectively. Using the Generalized Cross Validation (GCV) score to protect against overfitting, we trained a MARS model that had 7, 10 and 6 mono-, bi- and trivalent terms, respectively. We were able to identify a number of highly significant multivalent terms, suggesting that multivalency and cross talk among histone modifications may be widespread. However, we were surprised that both models predicted H4R3me2--shown to be repressive in a number of experimental studies [15-28]--to be among the most repressive histone methylations given that its ChIP-Seq enrichment levels showed no response to increasing gene expression [14].

## Methods

### Calculation of amplitudes

Enrichment levels, or amplitudes, for each of the 21 histone modifications were estimated for each gene using a spatially weighted average of the mapped ChIP-Seq tag counts (see Additional file 1: Supplemental Figure S1 and Additional file 2: Supplemental Table S1 for the range of amplitude values). The gene list used in this study was compiled from the NCBI36 *Homo sapiens *database (Ensembl 54, downloaded June 24, 2009). For each mark *j*, an average enrichment template, *t_i,j _*across the 5' flanking region (i.e., -2 kbp before the transcription start site), the body of a scaled gene (a gene divided into a fixed number of bins), and the 3' flanking region (i.e., transcription stop site to +2 kbp), was first calculated as a function of relative genomic position *i*. For both the 5' flanking region and 3' flanking region, the coordinate *i *represents each nucleotide position relative to the transcription start and stop sites, respectively. Within gene bodies (i.e., transcription start site to stop site), the coordinate *i *represents the position in the gene body, which is divided into 8138 segments, or bins, which corresponds to the median gene length. For genes whose lengths are greater than 8138 bp, tag counts were averaged across bases within each of the 8138 bins. For genes whose lengths are less than 8138 bp, tag counts were repeated in order to generate 8138 bins. For genes not divisible by 8138 or a divisor thereof, fractions of base pairs within a bin were rounded to the nearest integer value; therefore bins containing the majority of the fraction received the full tag count value of the corresponding base pair, while the bin containing the minority received no part of the bisected base pair's value. The median value was used for the bin number to minimize biases introduced in scaling. Large bins would tend to over-smooth large genes, while small bins would tend to overrepresent copied values from small genes. The *t_i,j _*or template for mark *j*, was finally computed by (1) aligning the transcription start and stop sites of every scaled gene and then (2) calculating the average bin-averaged tag count across genes for every coordinate *i*. All templates were then normalized so that their average across bins was 1 (; *N *= number of bins). In other words, the template is the averaged and normalized enrichment profile across all scaled genes. Because the template appears to have a characteristic shape for a given mark *j *across the length of scaled genes, we developed a model of relative enrichment which assumes the actual profile of any given mark is given by a product of a gene-dependent amplitude, , for a gene, *k*, and the mark's template *t_i,j_*. In other words, gene *k*'s tag count profile for mark *j *across genomic coordinate *i*, , is well approximated by . Using least squares, we minimized the difference between the model and the actual tag count profiles:(1)

to arrive at the following equation for mark *j*'s amplitude at gene *k*:(2)

We note that in the special case where the template is constant as a function of genomic position/bin, *i*,  reduces to a simple average of tag counts across bins, , which is the appropriate estimate of tag "depth" for a mark whose tag distribution is uniform across a gene.

### Selection of transcription start and stop sites

Many Ensembl genes contain multiple start and stop sites. Given that we only have 3' biased gene expression data, there are cases where we cannot unambiguously assign an Affymetrix probe set to one transcription start or stop site which we need for our estimate of mark enrichment. Consequently, we chose the transcription start sites that were associated with the highest number of significantly enriched histone modifications as representing the most likely expressed transcript. If a selected start site had multiple stop sites, we chose a stop site using the same scheme. In cases where multiple transcription start sites had the same number of significant marks, the most upstream transcription start site was chosen. When multiple stop sites for a given start site had the same number of significant marks a stop site was arbitrarily selected.

To determine the number of significantly enriched marks for a particular transcription start and stop site, we first calculated the distribution of mark amplitudes for all Ensembl genes. The left tail relative to the mode of the distribution of amplitudes for a particular mark was used to build a Gaussian null model as a background noise model for that mark. The mode of the amplitude distribution was used as the mean of the null model, and the standard deviation of the null model was derived using the following equation:(3)

where *μ*_*j *_is the mode of the amplitude distribution and the sum is over genes k* whose amplitude is less than or equal to the mode  and *n *is the number of genes that satisfy this inequality. This null model was used to determine the p-value by calculating the integral of the Gaussian from the mark amplitude to infinity for each mark at every Ensembl gene [29,30].

A Benjamini-Hochberg false discovery rate (FDR) [31] correction was applied to the p-values using the p.adjust function in R, and an FDR-corrected p-value cutoff of 0.05 was used to determine significantly enriched amplitudes.

### Amplitude robustness and relative error metrics

To assess the fit of our template model to the data we calculate the coefficient of variation of the root mean square deviation CV(RMSD) for every gene, which is defined by:(4)

where n is the number of bins in the template, and all other variables follow previous definitions. In addition to the gene amplitude calculation with the 8138-bin (8 k) template, amplitudes were also calculated with 6000 (6 k) and 10,000 (10 k) bin (plus flanking regions) templates. To assess the robustness of our amplitude estimates, we calculated the Spearman correlation coefficient between the 8 k and 6 k bin amplitudes and the 8 k and 10 k bin amplitudes. We also calculated the fractional difference between the 8 k and 6 k bin amplitudes, , and the 8 k and 10 k bin amplitudes, which is similarly defined. Finally, the CV(RMSD) was calculated for all marks for the 3 sets of amplitude calculations to assess effect of bin size selection on the template model fit.

### Building the multilinear model using stepwise linear regression

We built the multilinear model using a stepwise linear regression procedure (*stepwisefit *in MATLAB), which models gene expression as a function of histone mark enrichment according to the following equation:(5)

where *Y*_*k *_is the normalized log_2 _gene expression (using GCRMA [32]); *β_j _*, *β_j,l _*, *β_j,l,m _*are mono-, bi- and trivalent histone modification fitting coefficients;  are mark *j *amplitudes for gene *k *and the *ε*^*k *^are random errors. Briefly, an initial model with randomly selected terms-defined as β coefficients multiplied by one, two or three mark amplitudes (e.g., )-- is fit. Terms from the set that are not in the initial model and make a statistically significant contribution to the model (i.e., p-value ≤ 0.05 according to an F-test) are added during a forward step. The forward step continues until no terms from the available pool of unused terms contribute significantly to the model. A backward step is then applied whereby terms are ranked in descending order according to their p-values and removed if they are not significant (i.e., p-value > 0.05). The backward step ends when no terms in the model are insignificant. The forward and backward steps are repeated until no significant terms can be added or removed, respectively.

Because stepwise linear regression is not guaranteed to converge to a globally optimal solution (e.g., minimum adjusted R^2^) for any given initial seed model, we performed multiple rounds of multiple stepwise regressions using different randomly seeded models. In the first round, we ran *stepwisefit *on the full data set 100 times using randomly seeded models. This resulted in 100 models with a mean of 227 terms. To assess the statistical significance of a given term's survival rate across the models, we randomly sampled 227 of the 1561 possible terms to generate a null model. While a survival rate of 0.2 was significant (p-value < 0.05), to increase stringency we arbitrarily selected a cutoff of 0.35 to arrive at 167 starting terms for the next round of stepwise linear regression.

To avoid the problem of overfitting and its inflation of model complexity, we applied *stepwisefit *to 10-fold cross validation data. Specifically, for each of the 10 folds we performed 10 runs of *stepwisefit *where the initial model contained the 167 terms found in the first round plus an additional 60 randomly selected terms (i.e., we generated 100 models). Using the test data, we applied only the backward step of the stepwise procedure to assess the significance of every term that survived the training step and removed those with p-values > 0.05. Among the 10 runs for each fold, the model with the lowest test mean square error (MSE) was selected. This resulted in 10 models for each fold. We then required a term to appear in 5 or more of the 10 models generated within each fold to be selected for the final model. This resulted in 24 terms.

We arrived at a robust estimate of the final set of 24 coefficients by fitting the training data to a model that contained only the 24 terms. This yielded 10 sets of 24 coefficients (i.e., one for each fold of the 10-fold training data). We arrived at the final value of each fitting coefficient by calculating the trimmed mean of the 10 found in each fold. The final model's performance was assessed by calculating the mean MSE and adjusted R^2 ^across the 10 test and training data folds (see Results and Discussion).

### Building the Multivariate Adaptive Regression Splines (MARS) model

The relationship between gene expression level and each mark's average enrichment tends to be nonlinear including saturation of gene expression response as a function of mark level. The "*earth*" package in R was used to build the MARS model, which naturally accounts for non-linear responses between the input and output variables. Briefly, a MARS model is the sum of basis functions multiplied by a coefficient to be determined from a regression analysis of the function(6)

where *Y*^*k *^is log_2 _gene expression of gene *k *(i.e., output variable), *c*_0 _is a constant,  is the subset of mark amplitudes that appear in term *i*, and *b_i_*(·) is a basis function that is made up of either one or a product of two or more hinge functions. Hinge functions are splines that take the form  or  where  is a special constant known as a *knot*. We note that the two hinge functions shown above are a symmetrical pair about the vertical line .

The MARS model was built in one forward and one reverse pass. The forward pass builds the model using a greedy algorithm. It begins with an intercept term that is equivalent to the mean of the observed response variable, which is log_2 _gene expression level in our case. The algorithm then searches for the monovalent contributions fitted as a pair of symmetrical basis functions, which maximally reduce the residual sum-of-squares (RSS) at each step. It then adds bivalent terms, which are constrained to contain one of the monovalent terms and maximally reduce the RSS at a given step until a minimum RSS reduction is reached. It adds trivalent terms, which are constrained to contain one of the bivalent terms and maximally reduce the RSS at a given step until a minimum RSS reduction is reached. The reverse pass then prevents overfitting by removing terms to optimize a Generalized Cross Validation (GCV) score. The GCV penalizes model complexity by dividing the RSS by the effective number of degrees of freedom in the model(7)

where *RSS *is the residual sum of squares, *N_g _*is the number of observations or genes with expression data in our case, *T *is the total number of terms in the model, *P *is a user defined penalty (*earth*'s default is 3 for multivalent models), and *B *is the total number of non-constant basis functions in the model.

## Results and discussion

### Analysis workflow

**Figure 1:**
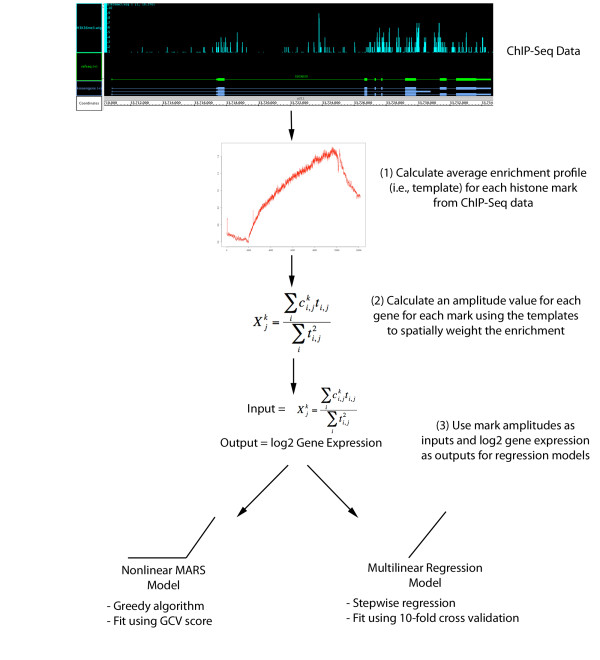
**Flowchart of multilinear and MARS model construction**. Chart describes the analysis steps in model construction. Starting with histone mark/variant ChIP-Seq data, template profile and amplitude calculation, and finally construction of regression models using mark amplitudes as inputs and log_2 _gene expression as outputs.

A diagram of our analysis workflow is shown in Figure 1. The inputs to our analysis are the read enrichment profiles across the human genome generated by Barski *et al. *[14]. In Figure two of their paper, they also calculated composite plots where they stratified gene expression by quartiles, aligned the transcription start sites (TSS) of all the genes and calculated the normalized read counts as a function of position relative to the TSS. These plots reveal that (1) each mark has a relatively unique profile and (2) the shape of each mark's profile displays a relatively weak dependence on gene expression level. Based on these observations, we modeled each modifications profile at every gene as a product of a gene dependent amplitude multiplied by a position dependent (i.e. relative to the TSS and transcription stop) average profile or template. From the read enrichment profiles, we calculated the average spatial distribution of each mark across the promoter region, the scaled gene and 3' of the transcription stop as detailed in the Methods section. We then calculate all possible 210 bivalent products and all possible 1330 trivalent products from the 21 single mark amplitudes. Thus, we have 21 single modification states that are inputs to the MARS model and a total of 1561 possible modification states that are inputs to our multilinear model.

These amplitudes were calculated for 11,796 Ensembl genes [33]. Because the gene expression data was 3' biased, we could not distinguish the expression levels of different isoforms, which included multiple TSS. We selected the TSS that had the largest number of significant modifications as detailed in the Methods section. Human CD4^+ ^T-cell gene expression data which was used to generate the output of our models was collected from the Genomics Institute of the Novartis Research Foundation's SymAtlas [34]--a compendium of gene expression data in human and mouse tissues. Only genes that had Ensembl, UCSC, and RefSeq IDs were included in this study. Of the 18,647 genes that met these criteria, 11,796 had expression data [34] associated with them. Because multiple Affymetrix probes can interrogate a single gene, the total number of expression data points for the 11,796 genes was 17,635, which constituted the output of the multilinear and MARS models.

### Estimating input amplitudes for regression models

Two groups [35,36] have estimated ChIP-Seq histone modification/variant enrichment levels across genes in order to applying machine learning techniques--linear regression [36] and Bayesian networks [35]. They count tags only in a region surrounding the transcription start site (i.e., ±1 kbp [35] or ±2 kbp [36] of the TSS). A major problem with this method is that many marks do not have promoter/5' end biased enrichment patterns. A striking example is H3K36me3, which has increasing enrichment along the gene body, which peaks near the 3' end of genes [14]. Yu *et al. *[28] calculate correlation coefficients between their 5' end biased mark enrichment estimates and gene expression levels and find little to no correlation between H3K36me3 and gene expression. This is an unexpected result as H3K36me3 has been characterized as an activating mark in a number of biochemical studies [7,8], and its levels have been shown to have a strong positive correlation with gene expression [14]. This discrepancy is likely due to the 5' bias of their amplitude estimation method. To address this problem, we estimated the effective enrichment levels of each mark by calculating a weighted average across the whole gene and its flanking region as described in the Methods section. We use the average enrichment pattern across the flanking regions and body of scaled genes as an estimate of the weighting function. However, given the large variation in gene lengths, exon/intron number, and mark deposition patterns, we assessed the robustness and relative error of our amplitude estimation procedure.

### Robustness and relative error of mark amplitude estimates

Our amplitude estimation procedure is motivated by the observation that a number of histone methylations (e.g., H3K36me3, H4K20me1, H2BK5me1, etc) are pervasive across the body of genes and their enrichment patterns appear to scale with gene length. However, methyl groups are physically attached to histones whose octamer wraps 146 bp of DNA (i.e., a nucleosome). Thus, our (and others [14]) gene-scaling procedures average different numbers of nucleosomes depending on the gene length and selected bin size. Consequently, we assessed the robustness of our amplitude estimation procedure by recalculating our template and amplitude using 6000 (6 k) and 10,000 (10 k) bins and compared them to those calculated using 8138 (8 k) bins.

We first generated scatter plots of the 6 k versus 8 k and 10 k versus 8 k amplitude estimates (Additional file 3: Supplemental Figure S2) and calculated their associated Spearman correlation coefficients (Additional file 4: Supplemental Table S2 and Additional file 5: Supplemental Table S3) for all 21 histone modifications/variants. We found the values to be highly correlated with correlation coefficients ranging from 0.994-0.9995 and 0.9975-0.9998 across marks for the comparisons of 8 k bins to 6 k and 10 k, respectively. We also calculated the fractional difference (i.e., difference divided by mean) between 6 k and 8 k and 10 k and 8 k amplitude estimates. These values were summarized across genes by calculating the 0^th^, 25^th^, 50^th^, 75^th ^and 100^th ^percentile values for each mark (Additional file 4: Supplemental Table S2 and Additional file 5: Supplemental Table S3). The absolute value of typical (50^th ^percentile) fractional differences range from 0.0023-0.065 and 0.0016-0.042 for the comparisons of 8 k to 6 k and 10 k, respectively. Indeed, the worst absolute values were 0.22 and 0.16 for 8 k versus 6 k and 10 k, respectively. Thus, our estimates of mark enrichment amplitudes are relatively robust with respect to bin size. Given these results, it's not surprising that our model results and main conclusions do not depend on bin size as discussed below.

An advantage of a model-based approach to estimating enrichment levels is that we can directly assess model performance by calculating residuals (i.e., differences between the model and the data). Thus, for each mark, we calculated the root mean square deviation (RMSD) between the model and the data divided by the amplitude (i.e., CV(RMSD) defined in Methods section) for every gene, which is a measure of the relative error. In a plot of CV(RMSD) versus amplitude for every mark we find a near universal curve (Additional file 6: Supplemental Figure S3). This results in part because our normalization of each mark's template (i.e., their average across bins equals 1) allows us to interpret the mark amplitude as a model-based estimate of each mark's effective read density, or read coverage. As might be expected, below amplitude values of 1 (i.e., 1× coverage) the error grows rapidly. For relatively large amplitudes (i.e., values greater than 1), the CV asymptotically reach values slightly below 2. In contrast, marks whose largest amplitudes fall well below 1 and consequently don't achieve their large amplitude asymptotic value have CV values that range from 2.3-5 at the largest amplitudes encountered (i.e., 95^th ^amplitude percentile). For most marks, the 95^th ^amplitude percentile is below 1, indicating from our crude gene-centric measure of coverage/read density that the effective sequencing coverage might be low (see Additional file 7: Supplemental Table S4). We also see a steady trend upward in the CV--calculated in the neighborhood of the 95^th ^amplitude percentile (mean CV calculated for genes in the 92.5-97.5 amplitude percentile)--with decreasing 95^th ^percentile amplitude levels (see Additional file 7: Supplemental Table S4). Taken together, these results indicate that RMSD between the model and the data are on the same order as the amplitude. We also note that we find essentially the same CV(RMSD) values for 6 k and 10 k bins. We are currently working on improving estimates of mark enrichment levels from ChIP-Seq data and distinguishing the extent to which this modest to high variability is due to intrinsic noise or systematic biases introduced by our model assumptions, including how we scale genes. Nevertheless, we find our weighted average estimate is relatively robust and should capture enrichment level trends in histone modification/variant ChIP-Seq data reasonably well.

### Multilinear model

**Figure 2:**
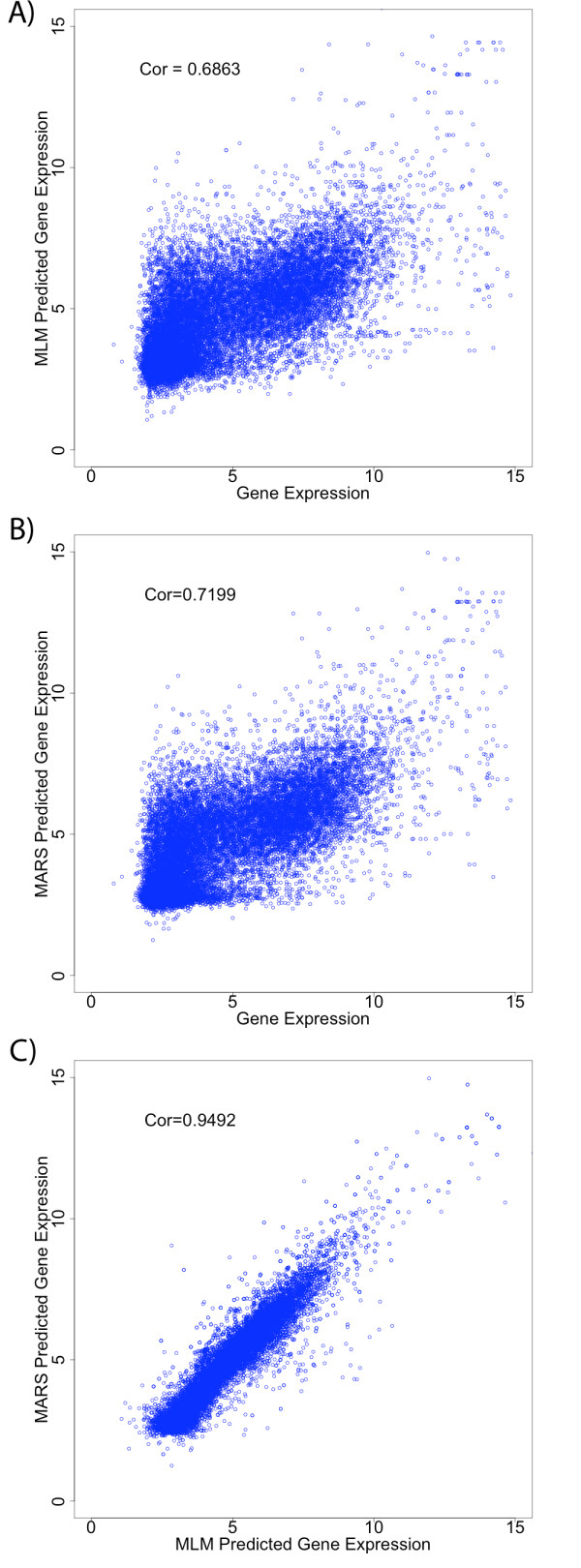
**Comparison of predicted and observed gene expression**. Scatter plots of (A) the multilinear model (MLM) predicted gene expression versus observed gene expression; (B) the MARS predicted gene expression versus observed gene expression; and (C) the MARS predicted gene expression versus multilinear model predicted gene expression. The corresponding Pearson correlation coefficient is shown within each plot.

We fit the gene expression data to the multilinear (ML) model shown in equation (5). As described in the Methods section, we used stepwise linear regression to build the ML model. There were 21, 210 and 1330 possible terms in the first, second and third sum of equation (5), respectively. The final model contained 24 terms. The average training and testing MSE was 3.1213 and 3.1525 respectively for this model. The average adjusted R^2 ^for the training and testing data is 0.4689 and 0.4574, respectively. The fact that the train and test values are close suggests that the model was not over trained. Using all the data, we calculated an adjusted R^2 ^of 0.4687 and MSE value of 3.1228. Crudely, this suggests that our model explains almost 50% of the gene expression variation after adjusting for the number of degrees of freedom. In Figure 2A, we show a scatter plot of the actual versus the model log_2 _gene expression levels whose associated Pearson correlation coefficient was 0.687 (p-value < 2.2e-16). This value is consistent with that of Karlic *et al. *[36] who modeled gene expression as a function of 38 histone methylation and acetylation modifications and H2A.Z using a linear model with no interaction (multivalent) terms. Given the absence of many other mRNA regulatory factors including other histone modifications, transcription factors, and miRNAs, a relatively significant percentage of the variation is explained by this model and the Karlic *et al. *model [36].

Finally, 7 of the 21 (33%) single terms were found to be significant in the model. In addition, there were 8 (4%) significant paired terms and 8 (0.6%) significant triplet terms. The terms appearing in the full model are displayed in Table 1 where we show each surviving term's β coefficient, the β coefficient's Z-score (i.e., the number of standard deviations away from β equaling 0; equation 3.12 of [13]), the term's p-value and a robust impact factor. The robust impact factor is defined as the product of the fitting coefficient (β) and the inter-quartile range (75^th ^percentile - 25^th ^percentile) of the mark amplitudes. It is a robust measure of a term's impact on gene expression while the Z-score and p-value are measures of its significance. A positive (negative) β coefficient, Z-score and impact factor indicate an activating (repressing) term in the model. The table is sorted by impact factor with activating and repressive marks labeled with an "a" and "r" superscript, respectively. We labeled the marks according to (1) the sign of their monovalent term in the ML model or (2) the response of the mark's levels with increasing gene expression when it did not contribute a monovalent term (these marks are starred).

**Table 1:** Multilinear model terms and statistics

Multilinear model term	β (trim mean)	Z (trim mean)	p (median)	Impact (trim mean)
**H3K79me1^a^**	6.741	18.234	0	1.331
**H3K36me3^a^**	4.087	17.802	0	0.922
**H3K79me3^a^**	3.078	23.916	0	0.598
**H4K20me1^a^**	0.977	21.446	0	0.450
H3K4me2^a^* - H3R2me1^r^	18.270	7.850	1.66E-15	0.437
H3K27me2^r^* - H3R2me1^r^	70.468	15.280	0	0.381
H3K9me2^r^* - H3K27me1^r^* - H4K20me1^a^	37.041	5.643	9.47E-09	0.156
**H3K4me3^a^**	0.133	5.729	5.20E-09	0.151
H2BK5me1^a^* - H3K36me3^a^	1.286	3.800	7.95E-05	0.115
H2BK5me1^a^* - H4K20me1^a ^- H3R2me1^r^	1.531	6.034	1.14E-09	0.030
**Intercept**	4.026	64.131	0	0
H3K9me3^r^* - H3K36me3^a^	-2.747	-12.439	0	-0.010
H3K4me2^a^* - H3K36me3^a ^- H3K79me3^a^	-1.274	-3.743	1.02E-04	-0.018
**H3K36me3^a ^- H3K79me2 - H3R2me2**	-5.855	-3.497	1.73E-04	-0.022
H3K27me3^r^* - H3K79me2 - H3K79me3^a^	-26.341	-6.380	5.78E-11	-0.026
H3K4me1^a^* - H3K9me2^r^* - H4K20me1^a^	-4.563	-3.578	2.65E-04	-0.041
H3K9me1^a^* - H3K27me1^r^* - H4K20me1^a^	-2.350	-5.078	1.92E-07	-0.077
H2BK5me1^a^* - H4K20me1^a^	-0.600	-9.627	0	-0.095
**H3K36me1 - H3K79me1^a ^- H3K79me3^a^**	-27.840	-8.478	0	-0.115
H3K4me2^a^* - H3K9me1^a^*	-1.578	-3.340	3.57E-04	-0.123
**H4R3me2^r^**	-11.121	-13.233	0	-0.301
H3K27me2^r^* - H3K36me3^a^	-31.772	-8.911	0	-0.311
H3K27me2^r^* - H3K79me1^a^	-56.535	-9.535	0	-0.449
**H3R2me1^r^**	-11.937	-16.504	0	-0.596

Terms appearing in the final multilinear model, and associated statistics. Trim mean of the β coefficients, Z-scores and impact factors (β multiplied by amplitude interquartile range). Trim mean is defined as the mean of the population excluding the lowest and highest 5% of the data. The superscript labels each mark as activating (a) or repressive (r). Unstarred marks correspond to monovalent terms in the model, starred marks do not have a monovalent contribution in the model, but correlated/anti-correlated with gene expression based on univariate analysis, and uncolored marks do not have clear correlation with gene expression. Rows are sorted by the impact term value.

### Multilinear model terms

Of the 7 monovalent terms (Table 1), H3K4me3, H3K36me3, H3K79me1, H3K79me3 and H4K20me1 were activating. Of these, only H3K4me3, H3K36me3 and H4K20me1 display a clear overall activating trend from composite plots [14]. Based on their composite plot analysis, Barski *et al. *conclude that H3K79me1 level alone shows no overall trend with gene expression while it makes the highest impact activating contribution in our ML model. This is consistent with a recent finding that H3K4me3 and H3K79me1 are the most predictive of gene expression levels in low CpG content promoters [36]. Barski *et al. *also find that H3K79me3 is enriched in active gene promoters, and in the body of silent genes. Two arginine methylations, H4R3me2 and H3R2me1, were the only repressive monovalent marks in the model. In contrast, Barski *et al. *[14] find no overall activating or repressing trend for these two methylations from their composite plot analysis. Marks that showed an activating trend from composite plots [14] but did not appear as monovalent terms in our ML model included H3K4me1,2, H3K9me1 and H2BK5me1. Marks that showed a repressive trend from composite plots but were absent at the monovalent level in our ML model were H3K27me2,3, and H3K9me2,3. H3K27me1 and the variant H2A.Z did not appear as monovalent terms in the ML model and displayed complex, non-monotonic enrichments as a function of increasing expression from their composite plots. Finally, marks that neither appeared at the monovalent level in the ML model nor showed any trend with respect to gene expression level were H3K79me2, H3R2me2 and H4K20me3.

While the majority of monovalent terms are activating, the majority of multivalent terms, 11 of 16, are repressive. Half of the 16 multivalent terms involve a mix of activating and repressing modifications according to either the sign of their monovalent term in the ML model or composite plot trends. This is interesting given the discovery of bivalent domains of H3K4me3 and H3K27me3. Indeed, two of the three highest impact and most significant repressive terms are bivalent. They both include H3K27me2 together with H3K79me1 and H3K36me3 respectively. The highest impact activating multivalent term is also bivalent and composed of an activating mark, H3K4me2, and a repressive mark, H3R2me1. Thus, at the bivalent level, the linear model terms suggest that there is significant overlap between opposing marks (i.e., activating and repressive) and that one of them tends to "override" the other, similar to the observation that H3K27me3 overrides H3K4me3 in ES cells [10,12].

**Figure 3:**
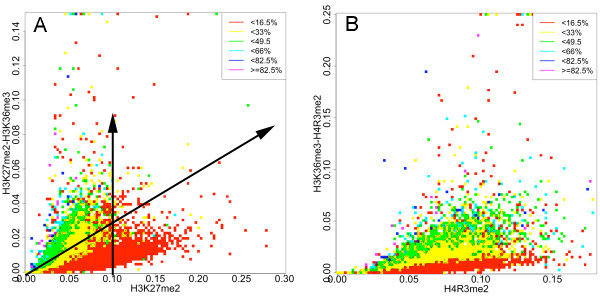
**Gene expression heat maps**. Heat maps of gene expression (color scale) as a function of bivalent (y-axis) and monovalent (x-axis) enrichment amplitudes for (A) H3K27me2-H3K36me3 versus H3K27me3 and (B) H3K36me3-H4R3me2 versus H4R3me2. The y-axis represents the product of the amplitudes of both marks and the x-axis represents one component of the pair. Gene expression values were binned into a 10,000 square grid with level represented by color. Vertical lines represent a constant value of the x-axis mark amplitude (i.e., H3K27me2 in (A) and H4R3me2 in (B)), while a line emanating from the origin represents a constant value of H3K36me3 in (A) and (B) with the slope corresponding to H3K36me3 level. Plot (A) shows mark avoidance, as there are few genes with high levels of both marks while (B) shows a trend toward mark concurrence. These plots also demonstrate how H3K36me3 strongly overrides H4R3me2 (increasing radial slope corresponds to increasing gene expression in (B)) but has more difficulty overriding the repressive activity of H3K27me2.

To further investigate the extent to which bivalent terms in the linear model point to the ability of one mark to override or oppose another overlapping mark, we generated heat maps, shown in Figure 3, of gene expression levels as a function of bivalent amplitudes (on the y-axis) and one of the monovalent amplitudes (on the x-axis). We discretize the amplitudes into a 10,000 square grid (i.e., 100 x-axis and y-axis bins) and calculate the average gene expression level within every box. The colors red, yellow, green, cyan, blue and magenta represent equidistant increasing gene expression values from the minimum to the maximum levels. As illustrated in Figure 3A, points along lines emanating from the origin moving outward represent increasing H3K27me2 (x-axis amplitude) and constant H3K36me3, with higher slopes corresponding to a higher level of fixed H3K36me3 amplitude. Conversely, points moving upward along vertical lines correspond to fixed H3K27me2 and increasing H3K36me3, with increasing position of the vertical line on the x-axis corresponding to higher level of fixed H3K27me2 amplitudes. This allows us to visualize a given modification's impact on transcription and how its regulation of transcription is continuously altered by the increasing co-occurrence of a second modification.

We find increasing H3K27me2 corresponds to decreasing expression as expected; we also find increasing expression with fixed H3K27me2 and increasing H3K36me3 (i.e., vertical lines at various x-axis intercepts). However, for relative H3K27me2 amplitudes exceeding 0.9, gene expression remains at low levels independent of H3K36me3 levels. This suggests that high levels of H3K27me2 are capable of overriding the gene activating potential of H3K36me3.

ML models have been applied in physical and statistical studies where a common outcome-- theoretically expected in many cases--is that the single terms dominate the model in both their relative impact and statistical significance. In these systems, the double, triple, quadruple product terms tend to make small, diminishing (in the order of the number of products) corrections to the single terms. As shown in Table 1, we find the expected trend with the highest impact and most significant activating terms all monovalent. The two most significant repressive terms are also monovalent: H3R2me1 (highest impact) and H4R3me2. However, we note that size of the impact of the highest impact bivalent terms is relatively large.

### MARS model

Normalized log_2 _gene expression was modeled as a function of histone modification enrichment using the nonlinear Multivariate Adaptive Regression Splines (MARS) method. The model was built with the *earth *package in R http://cran.r-project.org/web/packages/earth/index.html as described in the Methods section.

The MARS model contained 24 terms: 23 basis functions, and a constant. The MSE and R^2 ^for this model are 2.8387 and 0.5183, respectively. Figure 2B shows a scatter plot of the actual versus the MARS-predicted log_2 _gene expression levels, whose associated Pearson correlation coefficient is 0.7199. There were a total of 7 monovalent terms, with 5 unique single marks; 10 bivalent terms, with 7 unique pairs; and 6 trivalent terms, with 4 unique triplets. Table 2 displays each term's hinge functions, the term's fitting coefficient and the number of probe sets impacted by each term. The basis functions can often have a value of zero for a wide range of amplitudes; for these probe-sets, the basis function has no impact. Thus, the number of impacted probe-sets is a measure of the global impact of each term. We also directly assess the impact of each term on gene expression as discussed below.

**Table 2:** MARS model terms

Coefficient	Hinge function	Genes with non-Zero value
5.531222	1	17635
-10.31971	h(H3K27me2-0.0611382)	6892
1.325129	h(H3K79me3-0.0948497)	7218
-10.70662	h(0.0948497-H3K79me3)	10417
-58.18392	h(0.0611382-H3K27me2)	10743
-3.112329	h(0.559645-H4K20me1)	13551
-3.327909	h(0.545052-H3K36me3)	16872
15.2676	h(0.125391-H4R3me2)	17316
118.4095	h(H3K27me2-0.0611382)*h(H3K9me1-0.674218)	67
39.42698	h(H2BK5me1-1.49934)*h(0.0611382-H3K27me2)	106
-1.089521	h(0.545052-H3K36me3)*h(H4K20me1-0.673666)	3136
0.7436266	h(1.24429-H3K79me2)*h(H4K20me1-0.559645)	4055
47.71129	h(H3K79me1-0.055087)*h(0.0948497-H3K79me3)	4268
-258.7383	h(0.055087-H3K79me1)*h(0.0948497-H3K79me3)	6149
16.1397	h(H3K27me2-0.0611382)*h(0.674218-H3K9me1)	6825
1390.367	h(0.0611382-H3K27me2)*h(0.0913244-H3K27me3)	9906
3.235207	h(0.545052-H3K36me3)*h(0.673666-H4K20me1)	13736
-37.13981	h(0.438075-H3K36me3)*h(0.125391-H4R3me2)	15588
5.809395	h(1.49934-H2BK5me1)*h(0.0611382-H3K27me2)*h(H4K20me3-0.477337)	100
-100.9374	h(0.0611382-H3K27me2)*h(0.0913244-H3K27me3)*h(H4K20me1-3.93081)	203
-0.2791515	h(1.59266-H3K4me3)*h(1.24429-H3K79me2)*h(H4K20me1-0.559645)	2971
74.37772	h(0.545052-H3K36me3)*h(0.0625376-H3K79me1)*h(0.673666-H4K20me1)	6329
-237.028	h(0.0611382-H3K27me2)*h(0.0913244-H3K27me3)*h(3.93081-H4K20me1)	9703
90.39805	h(1.49934-H2BK5me1)*h(0.0611382-H3K27me2)*h(0.477337-H4K20me3)	10537

Coefficients and hinge functions within a row are multiplied and added to the products of other rows to form the MARS model. The rightmost column indicates the number of genes for which the hinge function takes a non-zero value. Terms appear in the order in which they were built into the model by the greedy algorithm.

### MARS model terms

Of the 5 unique monovalent marks in the MARS model, 3 are activating, including H3K36me3, H3K79me3 and H4K20me1. These results are in agreement with the ML model and the Barski *et al. *data; although as previously mentioned, H3K79me3 is a complicated mark, which is enriched in the promoters of active genes *and *the bodies of repressed genes. H3K27me2 has a repressive trend in the model, which agrees with Barski *et al. *H4R3me2 appears to have no discernible behavior in the Barski *et al. *data; however, both the MARS and ML models select it as a repressive monovalent mark.

**Figure 4:**
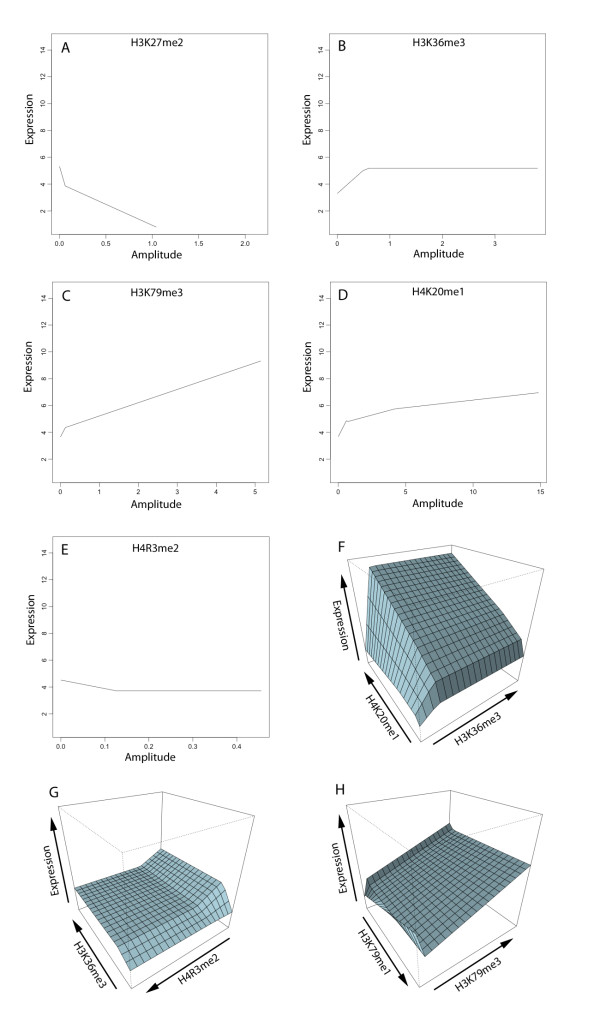
**MARS response plots**. Predicted gene expression versus amplitude for either one (2 D plots) or two marks (3 D plots) for (A) H3K27me2 (B) H3K36me3 (C) H3K79me3 (D) H4K20me1 (E) H4R3me2 (F) H4K20me1-H3K36me3 (G) H3K36me3-H4R3me2 and (H) H3K79me1-H3K79me3. Each axis represents the full range of expression and amplitude values. The trend of plots represents activating (positive slope) or repressive (negative slope) behavior. Many individual marks (A)-(E) and pairs (F)-(H) show some saturation effects and nonlinear behavior that could not be captured with a linear model; H3K36me3 (B), H4K20me1 (D) and H4R3me2 (E) show particularly distinct saturation effects. The combination H4K20me1-H3K36me3 (F) shows a dramatic nonlinear, synergistic activating effect. In contrast, the two marks in the combination H3K36me3-H4R3me2 (G) show opposing effects in that H3K36me3 activates and H4R3me2 represses gene expression.

The MARS model also shows nonlinear trends in log_2 _gene expression as a function of mark amplitude. Figure 4 shows plots of predicted log_2 _gene expression as a function of one or two mark amplitudes with all others fixed to their median value. These plots reveal whether a mark is activating or repressive. Not surprisingly, the dominant non-linear trend is saturation of predicted gene expression with increasing mark amplitude. This trend is clearly evident in both monovalent and bivalent plots shown in Figure 4.

To determine the global (full model) impact of individual marks appearing in the model, predictions were made with high enrichments (95^th ^percentile) and low enrichments (5^th ^percentile) of a given mark while fixing all other mark amplitudes at their median value. The difference of these predictions (Table 3--only non-zero values shown) provides an estimate of each individual mark's impact, where positive (negative) values correspond to activating (repressive) activity. This analysis shows general agreement with Barski *et al. *However, H2BK5me1, which does not appear as a monovalent term in the model, is activating in the Barski *et al. *analysis and repressive in the MARS model. Several marks that showed no activating or repressive trend in the Barski *et al. *analysis made significant contributions to the MARS model including, H3K79me1 (activating in the model), H4R3me2 (repressive), and H4K20me3 (repressive). H4K20me3 is generally associated with heterochromatin [8], possibly explaining why it has a slightly repressive trend in the MARS model. Interestingly, H4R3me2 has the second highest repressive impact, -0.45, and affects 100% of the probe-sets--the most of any term in the MARS model. It has been shown that DNA methylation, which is associated with gene silencing, is dependent on H4R3me2 [15].

**Table 3:** Impact of marks in MARS model

Mark	Predicted impact (95th-5th)
**H3K27me2**	-1.118
**H4R3me2**	-0.446
**H3K27me3**	-0.348
**H2BK5me1**	-0.281
**H4K20me3**	-0.055
**H3K79me1**	0.324
**H4K20me1**	1.473
**H3K79me3**	1.520
**H3K36me3**	1.650

The difference in mean predicted gene expression between the high (95^th ^percentile) and low (5^th ^percentile) amplitude values for a given mark while fixing all other mark amplitudes to their median values. Rows are sorted by predicted impact.

Based on the response plots (Figure 4), most marks appearing in a bivalent term seem to modulate each other modestly, with the exception of H4K20me1-H3K36me3, which shows complex synergistic, nonlinear behavior. Synergies were assessed by making model predictions while varying each of the unique interaction terms in the model. A model prediction was made with every combination of high enrichment (95^th ^percentile) and low enrichment (5^th ^percentile) for each element of a multivalent pair or triplet, while all other marks were held at their median values (Tables 4 and 5). The H4K20me1-H3K36me3 combination is an example of a strong synergistic, activating bivalent pair, where high levels of both correspond to highly active genes. The trivalent combination, H3K36me3-H3K79me1-H4K20me1, also shows strong synergistic activation, further suggesting that co-occupancy of H4K20me1 and H3K36me3 positively contributes to gene expression. Furthermore, this pair affects a large number of probe-sets, approximately 80% of those included in the model.

**Table 4:** Impact of two marks in MARS model

Bivalent MARS term	low-low	low-high	high-low	high-high
**H3K27me2-H3K9me1**	4.796	4.796	3.866	3.377
**H3K27me2-H3K27me3**	5.212	3.723	3.677	3.677
**H2BK5me1-H3K27me2**	5.009	3.677	3.807	3.677
**H3K36me3-H4R3me2**	3.530	3.468	5.618	4.556
**H3K79me2-H4K20me1**	3.709	5.211	3.709	4.879
**H3K79me1-H3K79me3**	3.009	5.938	4.721	5.228
**H3K36me3-H4K20me1**	3.217	4.163	4.599	7.218

The mean predicted gene expression using high (95^th ^percentile) and low (5^th ^percentile) amplitude values of each mark described in the leftmost column. The permutations of high and low values in each column correspond to the mark order in the leftmost column. The rows are sorted by the values in the last column.

**Table 5:** Impact of three marks in MARS model

Trivalent MARS term	low-low-low	low-low-high	low-high-low	low-high-high	high-low-low	high-low-high	high-high-low	high-high-high
**H2BK5me1**								
**H3K27me2**	5.087	4.827	3.677	3.677	3.845	3.719	3.677	3.677
**H4K20me3**								

**H3K27me2**								
**H3K27me3**	4.739	7.352	3.373	4.616	3.327	4.570	3.327	4.570
**H4K20me1**								

**H3K4me3**								
**H3K79me2**	3.709	4.944	3.709	4.722	3.709	5.712	3.709	5.172
**H4K20me1**								

**H3K36me3**								
**H3K79me1**	4.000	3.561	3.649	4.595	4.058	6.616	5.031	7.651
**H4K20me1**								

The mean predicted gene expression using high (95^th ^percentile) and low (5^th ^percentile) amplitude values of each mark described in the leftmost column. The permutations of high and low values in each column correspond to the mark order in the leftmost column. The rows are sorted by the values in the last column.

We also find one bivalent and trivalent combination composed of activating and repressive marks, H3K36me3-H4R3me2 and H3K27me2-H3K27me3-H4K20me1. As shown in Tables 4 and 5, we find that increasing each mark's level independently results in the expected activating or repressive response. High levels of the activating and repressive marks result in a moderating effect on predicted gene expression with values falling between those of high activating-low repressive and low activating-high repressive mark amplitudes. This reinforces the results of the ML model where we found the tendency of one mark to override or oppose another overlapping mark.

### Model comparison

Like the ML model, the MARS model explains about half of the variation in gene expression. Moreover, the ML and MARS model predicted gene expression profiles are highly correlated (Figure 2C). However, the Pearson correlation coefficient between predicted and actual log_2 _gene expression is slighter better for the MARS model. This is impressive given that both models contain the same number of terms, 24, and the MARS model was built using one round of a greedy algorithm while the ML model was built by selecting the best model from multiple rounds of a stepwise algorithm. We note that the stepwise algorithm is a more powerful and computationally expensive optimization procedure. These observations suggest that methods like MARS that are capable of modeling the nonlinear relationship between histone modification and gene expression levels should outperform models that assume this relationship is linear. Moreover, many of the bi- and trivalent terms in the ML model may not have a biological origin but are compensating for the nonlinearities in the data. Specifically, as shown in Table 1, the ML model contains two bivalent terms (H3K4me2-H3K9me1 and H2BK5me1-H4K20me1) containing activating marks with a negative (repressive) fitting coefficient, one bivalent term composed on two repressive marks (H3K27me2-H3R2me1) with a positive (activating) fitting coefficient, and a trivalent term composed of activating marks (H3K4me2-H3K36me3-H3K79me3) with a negative (repressive) fitting coefficient. These terms have no known biological origin and are more likely artifacts of imposing a linear model on data, which is inherently nonlinear.

Both regression methods produced a model with 24 terms. However, there are only 4 common terms between the models, all of which are monovalent terms: H4R3me2, H3K79me3, H4K20me1 and H3K36me3. Both models agree that of these marks H4R3me2 is the only repressive mark, while the others are activating. Considering the linear model contains 7 monovalent terms out of a possible 21, and the MARS model contains 5, the degree of overlap in the monovalent terms is quite high.

No overlapping multivalent terms existed between the two models. The differences between the multivalent components of each model could be the result of the way the models were built. Since the space of possible terms increases rapidly with valency, and the search space over which the ML model converges on a final model is much larger than that of the MARS model, the potential for model overlap becomes less likely as valency increases. In this way the MARS model--built using a greedy algorithm--is constrained in the number of multivalent terms it can potentially include.

### *In silico *knockout analysis

In order to generate experimentally testable predictions, a knockout analysis of the ML and MARS models was performed to assess the effect of removing a specific modification on gene expression. Predictions of gene expression were made with each model by setting the amplitude of a single mark to zero in the model and holding all others at their experimental values. This process was repeated for all marks in each model to determine the global effect of each histone modification. All pairwise combinations of marks were also knocked out for each model.

**Figure 5:**
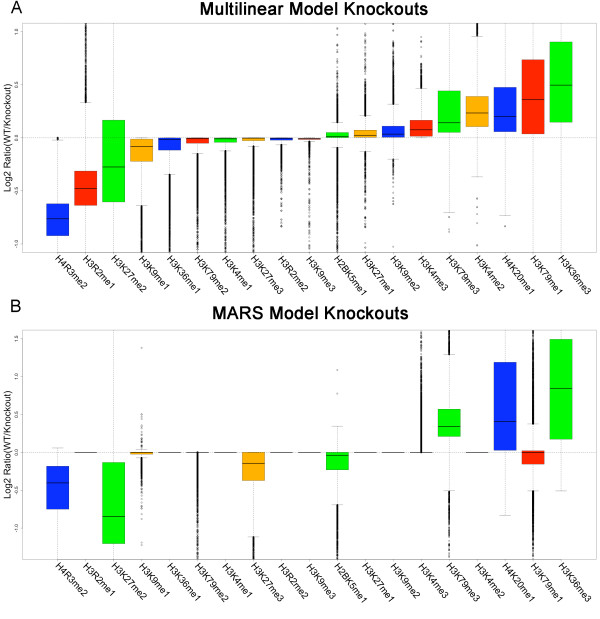
**Box plots of MLM and MARS knockouts**. Box plots representing the predicted log_2 _fold change (WT/KO) in gene expression after knocking out (setting mark amplitude to zero) a single mark while holding all other amplitudes at their experimental values in both the multilinear (A) and MARS (B) models. Negative shifts indicate repressive marks and positive shifts indicate activating marks. Both models show general agreement in knockout effects. Interestingly, both models choose H4R3me2 to be among the most globally repressive marks, whereas previous studies comparing H4R3me2 levels to gene expression have shown little to no correlation, suggesting the repressive character of H4R3me2 becomes apparent in a multivariate analysis of multiple modifications.

Tables 6 and 7 show the single and pairwise knockouts that have the highest impact on global gene expression in the ML and MARS models, respectively, where knockouts are represented as log_2 _fold changes of wild type over knockout predictions; positive values indicate activating marks, and negative values indicate repressive marks. Both sets of knockouts identify H3K36me3 and H4K20me1 to be among the strongest global activating marks, and H4R3me2 and H3K27me2 to be among the strongest global repressive marks. They also indicate that combinations of H3K79 methylations, H3K36me3 and H4K20me1 are among the strongest pairwise global activating marks, while combinations of H3K27 methylations and H4R3me2 are among the strongest pairwise global repressive marks. Figure 5 shows box plots of the log_2 _fold changes for each single knockout of the ML and MARS models. The general trend of knockouts in both models is similar; however, the MARS model includes fewer marks, and thus many of the knockouts (trivially) have no effect. Most of the marks absent from the MARS model have a very modest knockout effect (median log_2 _expression ratio magnitude < 0.1) in the ML model with the exceptions of H3R2me1 and H3K4me2. We note that the results of the knockout analysis are also robust with respect to bin size (see Additional file 8: Supplemental Table S5).

**Table 6:** ML model knockout analysis

Multilinear model knockouts	log2 fold change (predicted WT/predicted KO)
**H4R3me2**	-0.782
**H3R2me1**	-0.394
**H3K27me2**	-0.235
**H3K9me1**	-0.183
**H3K4me3**	0.108
**H3K4me2**	0.285
**H3K79me3**	0.344
**H4K20me1**	0.359
**H3K79me1**	0.428
**H3K36me3**	0.546
**H3K27me2-H3R2me1**	-1.192
**H3R2me1-H4R3me2**	-1.175
**H3K27me2-H4R3me2**	-1.017
**H3K9me1-H4R3me2**	-0.964
**H3K36me1-H4R3me2**	-0.912
**H3K79me2-H4R3me2**	-0.859
**H3K27me3-H4R3me2**	-0.837
**H3K4me2-H3K36me3**	0.857
**H3K79me1-H3K79me3**	0.903
**H3K36me3-H4K20me1**	0.907
**H3K36me3-H3K79me3**	0.916
**H3K36me3-H3K79me1**	0.976

The log_2 _fold change (predicted WT/predicted KO) in average gene expression for single and double knockouts in the multilinear model. *In silico *knockouts were performed by setting mark amplitudes to zero while fixing all other marks at their experimental values and making model predictions for each gene. The top 5 most repressive and activating fold changes for single and double knockouts are shown. Rows are sorted according to log_2 _fold change for single and double knockouts separately.

**Table 7:** MARS model knockout analysis

MARS model knockouts	log2 fold change (predicted WT/predicted KO)
**H3K27me2**	-0.742
**H4R3me2**	-0.506
**H3K27me3**	-0.244
**H2BK5me1**	-0.158
**H3K79me2**	-0.046
**H3K4me3**	0.054
**H3K79me3**	0.421
**H4K20me1**	0.715
**H3K36me3**	0.941
**H3K27me2-H3K27me3**	-2.333
**H2BK5me1-H3K27me2**	-1.329
**H3K27me2-H4R3me2**	-1.248
**H3K27me2-H4K20me3**	-0.973
**H3K27me2-H3K79me2**	-0.789
**H3K36me3-H3K4me3**	0.996
**H3K36me3-H4R3me2**	1.011
**H3K79me3-H4K20me1**	1.136
**H3K36me3-H4K20me1**	1.327
**H3K36me3-H3K79me3**	1.362
**H3K79me1-H3K79me3**	1.553

The log_2 _fold changes (predicted WT/predicted KO) in average gene expression for single and double knockouts in the MARS model. *In silico *knockouts were performed by setting mark amplitudes to zero while fixing all other marks at their experimental values and making model predictions for each gene. The top 5 most repressive and 4 activating fold changes for single as well as the top 5 most repressive and activating double knockouts are shown. Rows are sorted according to log_2 _fold change for single and double knockouts separately.

### H4R3me2 is globally repressive in ML and MARS models

Strikingly, H4R3me2 was the most globally repressive mark in the ML model according to the knockout analysis, with an average predicted fold change (WT/knockout) in gene expression of 0.55. It was also the second most repressive mark in the MARS model knockout analysis, with an average fold change of 0.70. This was a highly unexpected result for H4R3me2 given the unresponsiveness of its ChIP-Seq enrichment levels to increasing gene expression [14]. Indeed, we used the data generated by Barski *et al. *but came to diametrically opposed conclusions regarding H4R3me2's influence on gene expression. Moreover, this conclusion is not altered by the selection of bin size, as H4R3me2 is the most highly repressive mark in the 6 k bin-based MARS model, and second most repressive in the 8 k and 10 k bin-based MARS models (Additional file 8: Supplemental Table S5).

In order to make sense of the apparently contradictory behavior of H4R3me2, we first note that we performed two analyses that differed from the Barski *et al. *[14] analysis: (1) estimated mark amplitudes using a model based weighted average and (2) modeled gene expression using all histone methylation amplitudes as input to the ML and MARS models. A trivial explanation would be that our amplitude estimation procedure now yields a response of H4R3me2 levels to gene expression, which is then reflected in the ML and MARS fitting coefficients. We directly tested this by generating boxplots of our mark amplitudes stratified by quartiles of gene expression shown in Additional file 9: Supplemental Figure S4. Consistent with Barski's [14] composite plots, we observe little to no response of H4R3me2 amplitudes with increasing gene expression. For comparison, we generate the same boxplots for H3K27me2 and observe a dramatic decrease in its amplitude with increasing gene expression level as expected. Thus, we can rule out the mark amplitude estimation procedure as an explanation. We are left with the interesting result that H4R3me2's repression of gene expression is revealed by analyses such as MARS and ML modeling which account for the simultaneous impact of the other histone methylations. In other words, the impact of any given histone methylation on gene expression is analyzed in the context of 20 other activating and repressive modifications.

In order to better understand H4R3me2's affect on gene expression, we performed a comparative analysis with H3K27me2, which is highly repressive in the ML and MARS model knockout analysis as well as the Barski *et al. *composite plot analysis. Specifically, we divided the ML model log_2 _fold changes of wild type over H4R3me2 and H3K27me2 knockouts by quintiles and calculated boxplots of predicted gene expression in the WT and KO cases as shown in Additional file10: Supplemental Figures S5A and S5B, respectively. The first 20% of the data (QU1) represents the genes most up regulated by knocking out the mark (i.e., the largest de-repression of gene expression). The last 20% of the data (QU5) represents the genes least up regulated by knocking out H4R3me2 and genes down regulated by knocking out H3K27me2. The trends in WT and KO gene expression across the stratified data are opposite for H4R3me2 and K3K27me2. For H4R3me2, the median log_2 _gene expression in the WT is relatively low, 4, in QU1 and increases slightly to 4.7 in QU5 with the knockout showing a similar trend (Additional file 10: Supplemental Figure S5A). In contrast, the H3K27me2 WT median starts out considerably higher in QU1, 5.9, and plummets 8-fold to 2.9 in QU5 with the knockout again showing a similar trend (Additional file 10: Supplemental Figure S5B). Thus, H3R3me2 tends to be consistently acting on relatively low expressed genes, and its removal is predicted to increase their expression ~1.7-fold on average. H3K27me2, on the other hand, has the highest impact, from our knockout analysis, on middle to high expressed genes and the least on silenced genes.

**Figure 6:**
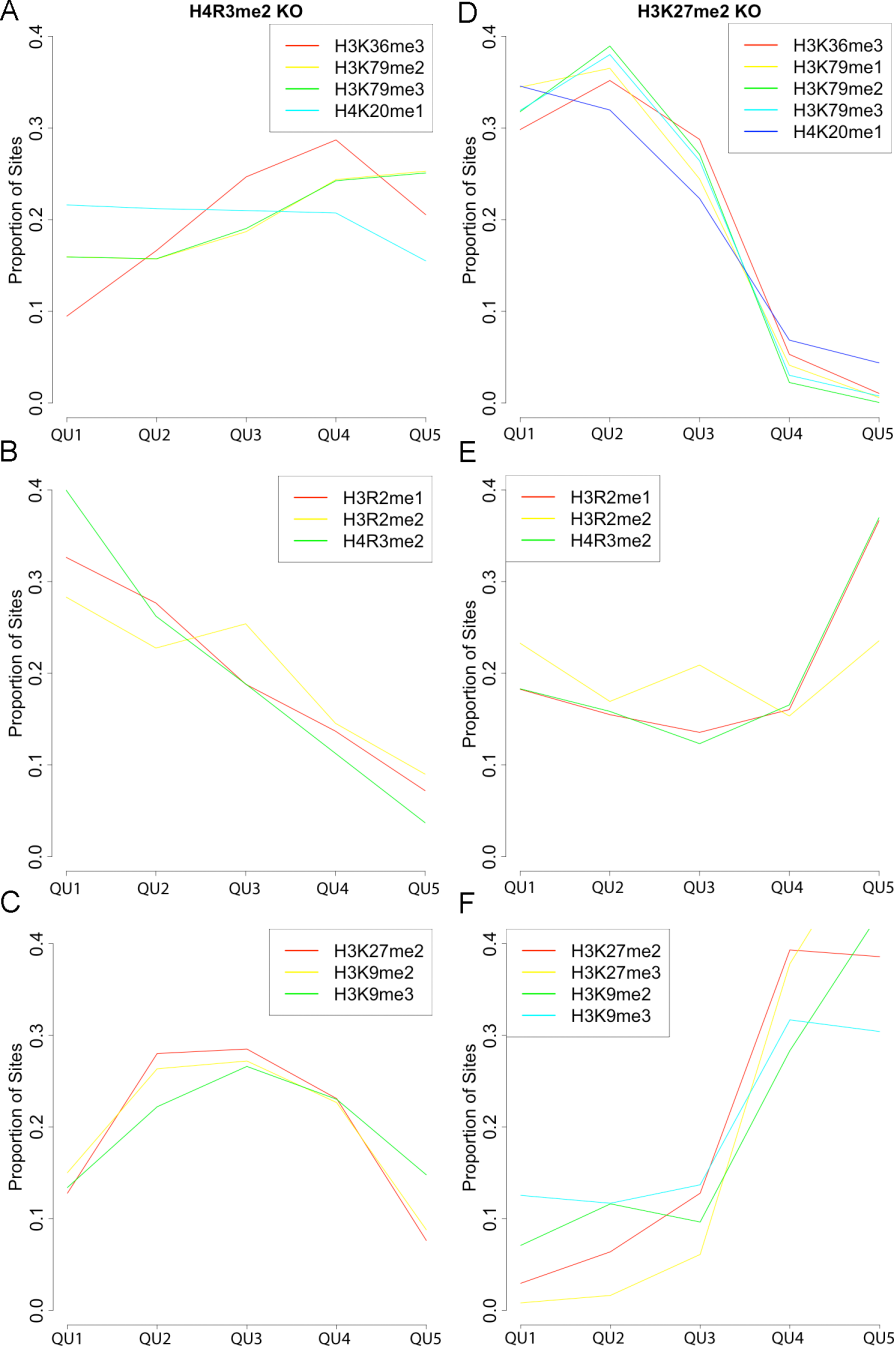
**Enriched sites across MLM knockout quintiles**. Plots show the proportion of significantly enriched sites identified by MACS (y-axis) for marks shown in the legend across the data divided by quintiles of log_2 _fold change (WT/KO) in gene expression predicted by the MLM for H4R3me2, (A)-(C), and H3K27me2, (D)-(F), knockouts. Proportions of sites were clustered using k-means clustering. For both knockouts activating marks clustered together, (A) and (D), as did arginine methylations, (B) and (E), and repressive marks, (C) and (F). H4R3me2 knockout effect only shows a strong correlation with other arginine methylations (B), while the H3K27me3 knockout effect shows strong anti-correlation with the activating marks (A) and strong positive correlation with other repressive marks (F).

We also calculated the proportion of significantly enriched marks (using MACS; see Methods) found in quintile-stratified log_2 _fold change data as shown in Figure 6A-F. Interestingly, the profiles of mark site proportions across the stratified log_2 _fold change clustered into activating marks (Figures 6A,D), arginine methylations (Figures 6B,E) and repressive marks (Figures 6C,F). As expected, we find that the proportion of H4R3me2 sites is highest in the highest impact knockout log_2 _fold change group (QU1) and monotonically decreases across subsequent groups (Figure 6B). The activating marks site profiles (Figure 6A) tend to be relatively low and flat or mildly increasing with decreasing log_2 _fold change. The repressive mark site profiles tends to start low, rise and then fall with decreasing log_2 _fold change (Figure 6C). Thus, arginine methylation itself appears to drive the impact of H4R3me2 knockout on gene expression. In stark contrast, the proportion of H3K27me2 and other repressive mark sites increase monotonically with decreasing impact of the H3K27me2 knockout on gene expression (Figure 6F) with the activating marks showing the exact opposite trend (Figure 6D). Again the arginine methylation proportion of sites cluster together and show a distinct profile falling and then rising with decreasing log_2 _fold change (Figure 6E). Interestingly, we find the largest impact of knocking out H3K27me2 tends to be in genes where H3K27me2 levels are relatively low and activating mark levels are relatively high. For these genes, H3K27me2 appears to be modulating or reducing gene expression from high to moderate levels (Additional file 10: Supplemental Figure S5B).

Taken together these analyses suggest that in contrast to well-characterized repressive modifications including H3K27 and H3K9 methylation whose levels tend to be strongly anti-correlated to activating modification levels (see Figure 3A), H4R3me2 and the other arginine methylations tend to be somewhat uncorrelated to both. Consequently, its absence at genes does not imply the presence of activating marks and high levels of expression (see Figure 3B). Conversely, high levels of H4R3me2 can coincide with modest to relatively high levels of activating marks like H3K36me3, which tend to override H4R3me2 (see Table 4, Table 7 and Figure 3B). Thus, its levels show no strong trend with increasing overall gene expression as Barski *et al. *found. Instead, dimethylation of H4R3 consistently tends to further repress low to modestly expressed genes nearly 2-fold on average, leading to relatively strong predicted de-repression in the ML and MARS models when knocked out.

### Experimental studies demonstrate H4R3me2 represses gene expression

Barski *et al. *[14] report that they used the antibody that recognizes symmetric dimethylated H4R3 (H4R3me2s), which is deposited by the arginine methyltransferase PRMT5. A number of experimental studies have shown that PRMT5 and H4R3me2 s repress gene expression [15-28]. In an experiment that is a direct analogue of our knockout analysis, silencing of PRMT5 in mouse cell lines resulted in more de-repressed than repressed genes in a microarray analysis [22], supporting our result that H4R3me2 s is globally repressive. PRMT5 is a member of the multi-subunit mSin3A and NuRD histone deacetylase complexes [26], suggesting H4R3me2 is associated with deacetylation and hence gene inactivation [22,23,26]. Interestingly, both the mSin3A-PRMT5 containing complex and recombinant PRMT5 methylate H4R3 and show an *in vitro *preference for methylating hypo-versus hyperacetylated histone H4R3 [26]. PRMT5 was also shown to interact with the MBD2/NuRD complex and that PRMT5 and MBD2 are recruited to CpG islands in a methylation-dependent manner with H4R3 methylated at these loci [25]. These results are consistent with our finding that H4R3me2 tends to further repress modest to low expressed genes, which are likely hypoacetylated, contain methylated CpG islands in their promoter regions or both.

In a recent study, H4R3me2 s was shown to be required for subsequent DNA methylation [15]--a repressor of gene expression. Indeed, H4R3me2 s was shown to be a direct binding target of the DNA methyltransferase DNMT3A [15]. Loss of H3R3me2 s through shRNA knockdown of PRMT5 resulted in reduced DNMT3A binding, loss of DNA methylation and six-fold induction of the fetal (γ) globin gene [15].

## Conclusions

Current genomic strategies for assessing whether a particular histone modification is activating or repressive involve (1) mapping it to the genome using ChIP-chip or ChIP-Seq and either (2) comparing boxplots of gene expression of genes with and without the mark [37] or (3) generating composite plots of average mark levels of genes stratified by gene expression level as in the Barski *et al. *[14] analysis. Using this approach, Barski *et al. *concluded that H4R3me2 is neither activating nor repressive because its levels showed no response with increasing gene expression level. Using the Barski *et al. *ChIP-Seq data of 20 histone lysine and arginine methylations and histone variant H2A.Z in CD4^+ ^T-cells, we built models of gene expression as a function of histone modification/variant levels using Multilinear (ML) Regression and Multivariate Adaptive Regression Splines (MARS). The response of monovalent (non-interacting) terms in the ML and MARS model indicate whether a given modification is activating or repressive. For most of the 20 histone methylations, our assignments agree with that of Barski *et al. *However, according to our *in-silico *equivalent of knocking out modifications, H4R3me2 is predicted to be the most and second most globally repressive histone methylation among the 20 studied in the ML and MARS models, respectively. A number of experimental studies show that PRMT5-catalyzed symmetric dimethylation of H4R3 is associated with repression of gene expression [15-28]. This includes a recent study, which demonstrated that H4R3me2 is required for DNMT3A-mediated DNA methylation [15]--a known global repressor of gene expression. Consequently, our study serves as the first demonstration that H4R3me2 represses gene expression using genomic data and shows that the regulatory role of some modifications like H4R3me2 can only be revealed by approaches that simultaneously analyze multiple activating and repressive modifications. Our findings point to a disconnect between traditional biochemical (e.g., silencing) and genomic approaches in assessing the activating or repressive potential of an individual modification. Indeed, assuming the biochemical studies were correct and H4R3me2 is repressive, one would conclude from the Barski *et al. *[14] analysis that the antibody they used for H4R3me2 did not work. Our results suggest that it worked extremely well. Taken together, our findings have broad implications for ChIP-Seq experimental design, analysis and interpretation and suggest an important role for retaining network level information in the analysis of ChIP-Seq data.

## Abbreviations

MARS: Multivariate Adaptive Regression Splines; MLM: Multilinear Model; ML: Multilinear.

## Authors' contributions

XX calculated the amplitudes, carried out the multilinear model analysis and helped draft the manuscript. SH carried out the MARS model analysis and helped draft the manuscript. MWM participated in study design and helped to draft the manuscript. SB conceived of the study, guided the analysis and drafted the manuscript. All authors read and approved the final manuscript.

## Supplementary Material

Additional file 1 **Figure S1. Box plots of enrichment amplitudes**. Box plots of estimated enrichment amplitudes for each of the 21 histone modifications. Click here for file

Additional file 2 **Table S1. Range of mark amplitudes**. The minimum, 5^th^, 25^th^, 50^th^, 75^th^, 95^th ^percentiles, and maximum amplitude values for each mark. Click here for file

Additional file 3 **Figure S2. Scatter plots of amplitude estimates**. Scatter plots between amplitudes calculated using different numbers of bins within scaled genes. Comparisons of the 6000 (6 k) bin versus 8138 (8 k) bin amplitudes are shown in (A)-(D), and 10,000 bin versus 8138 (8 k) bin comparisons are shown in (E)-(H). Four selected marks are shown: H3K27me2 in (A) and (E), H4R3me2 in (B) and (F), H3K4me3 in (C) and (G), and H3K36me3 in (D) and (H). The corresponding Spearman correlation coefficient (CC) is shown within each plot. Click here for file

Additional file 4 **Table S2. Amplitude estimation robustness, 6 k vs 8 k template**. For every mark, the Spearman correlation coefficients and 0^th^, 25^th^, 50^th^, 75^th^, and 100^th ^percentile fractional differences between amplitudes calculated with the 6 k bin and 8 k (8138) bin templates. Click here for file

Additional file 5 **Table S3. Amplitude estimation robustness 10 k vs 8 k**. For every mark, the Spearman correlation coefficients and 0^th^, 25^th^, 50^th^, 75^th^, and 100^th ^percentile fractional differences between amplitudes calculated with the 6 k bin and 8 k (8138) bin templates. Click here for file

Additional file 6 **Figure S3 Relative error of mark enrichment models**. CV(RMSD) versus amplitude. Colors represent different marks as shown in the legend. Low amplitudes correspond to low levels/coverage, and thus high CV(RMSD) values. As amplitude increases, values reach an asymptotic value. Click here for file

Additional file 7 **Table S4. Relative error of mark enrichment models**. For every mark, the large amplitude CV(RMSD) values--mean CV of 92.5-97.5 percentile amplitude genes--calculated using 6000, 8138 and 10,000 bin templates along with the corresponding 95^th ^percentile amplitudes. Rows are sorted by the 8138 bin 95^th ^percentile amplitudes. Click here for file

Additional file 8 **Table S5. MARS knockout robustness**. Two additional MARS models were built with amplitude estimations using 6000 and 10,000 bins for the scaled gene. The table shows a comparison of knockout analyses performed for each model, with the results sorted by log_2 _fold changes calculated from the 8138-bin model. Overall, the results are quite robust, showing the same trend in nearly every mark. Furthermore, H4R3me2 appears as the most or second most repressive mark in each model. Click here for file

Additional file 9 **Figure S4. Box plots of amplitudes across expression**. Box plots of H4R3me2 (A) and H3K27me2 (B) amplitudes across the data stratified by quartiles of gene expression, where Q1 and Q4 represent the lowest and highest gene expression groups, respectively. Click here for file

Additional file 10 **Figure S5. Box plots of predicted gene expression before and after knockout**. Box plots of predicted gene expression before and after knockout of (A) H4R3me2 and (B) H3K27me2. Plots are stratified along the x-axes by quintiles of log_2 _fold change (WT/KO) in gene expression predicted by the MLM. Click here for file
