# Supplementary figures and images for: Application of machine learning methods to histone methylation ChIP-Seq data reveals H4R3me2 globally represses gene expression
Source: BMC Bioinformatics. 2010 Jul 23;11:396. doi: 10.1186/1471-2105-11-396 (PMC2928206; doi:10.1186/1471-2105-11-396)

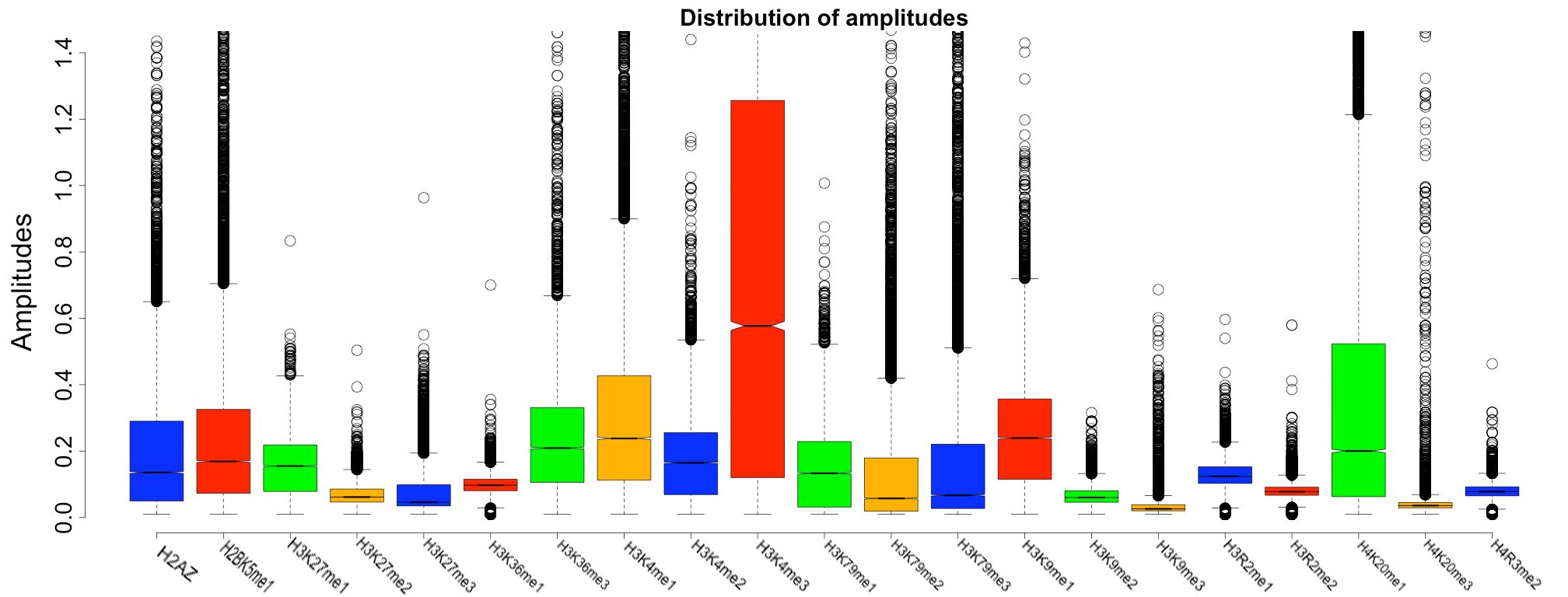

Supplement: Additional file 1 — Figure S1. Box plots of enrichment amplitudes. Box plots of estimated enrichment amplitudes for each of the 21 histone modifications. [file 1471-2105-11-396-S1.PNG]

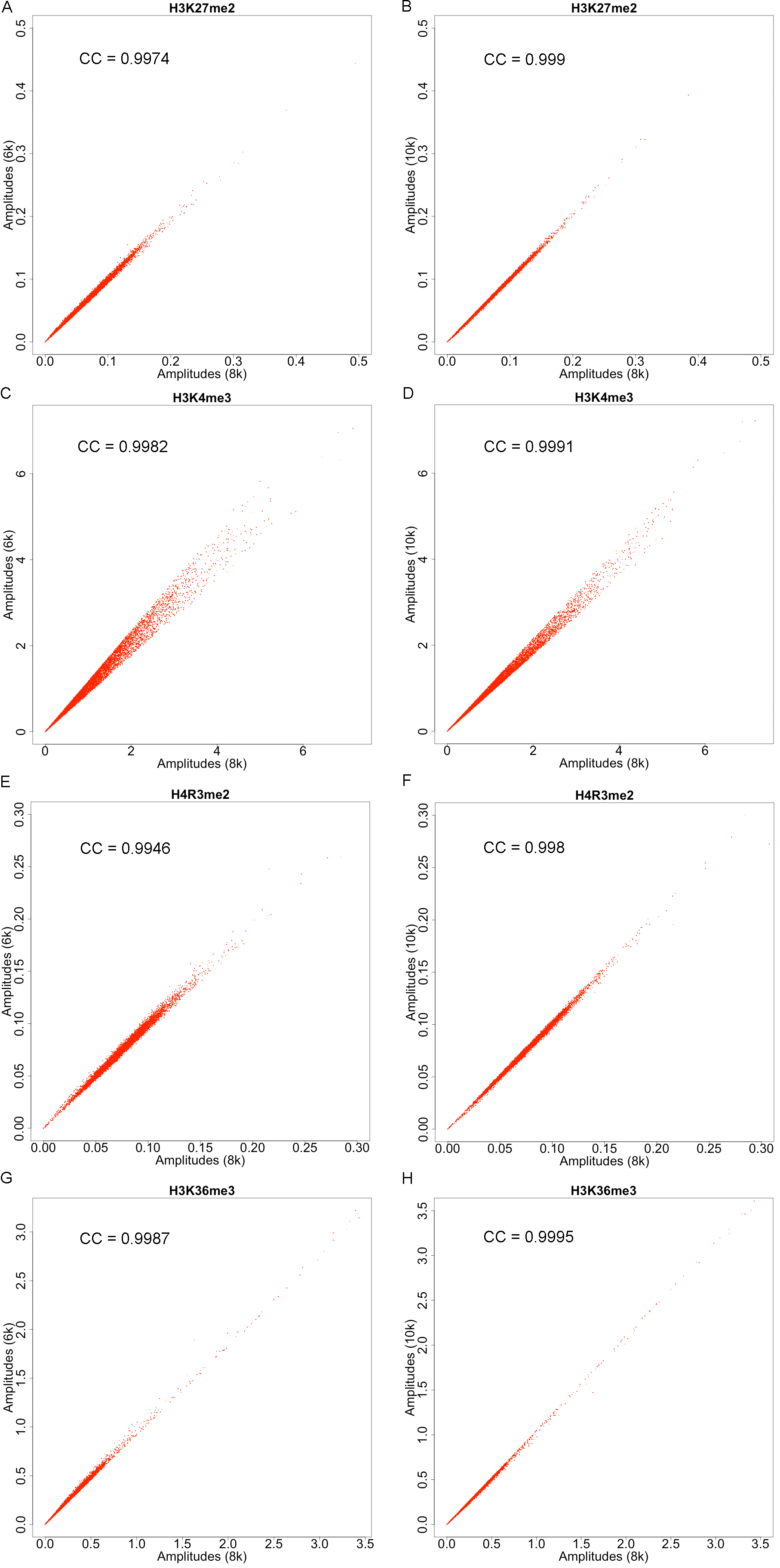

Supplement: Additional file 3 — Figure S2. Scatter plots of amplitude estimates. Scatter plots between amplitudes calculated using different numbers of bins within scaled genes. Comparisons of the 6000 (6 k) bin versus 8138 (8 k) bin amplitudes are shown in (A)-(D), and 10,000 bin versus 8138 (8 k) bin comparisons are shown in (E)-(H). Four selected marks are shown: H3K27me2 in (A) and (E), H4R3me2 in (B) and (F), H3K4me3 in (C) and (G), and H3K36me3 in (D) and (H). The corresponding Spearman correlation coefficient (CC) is shown within each plot. [file 1471-2105-11-396-S3.PNG]

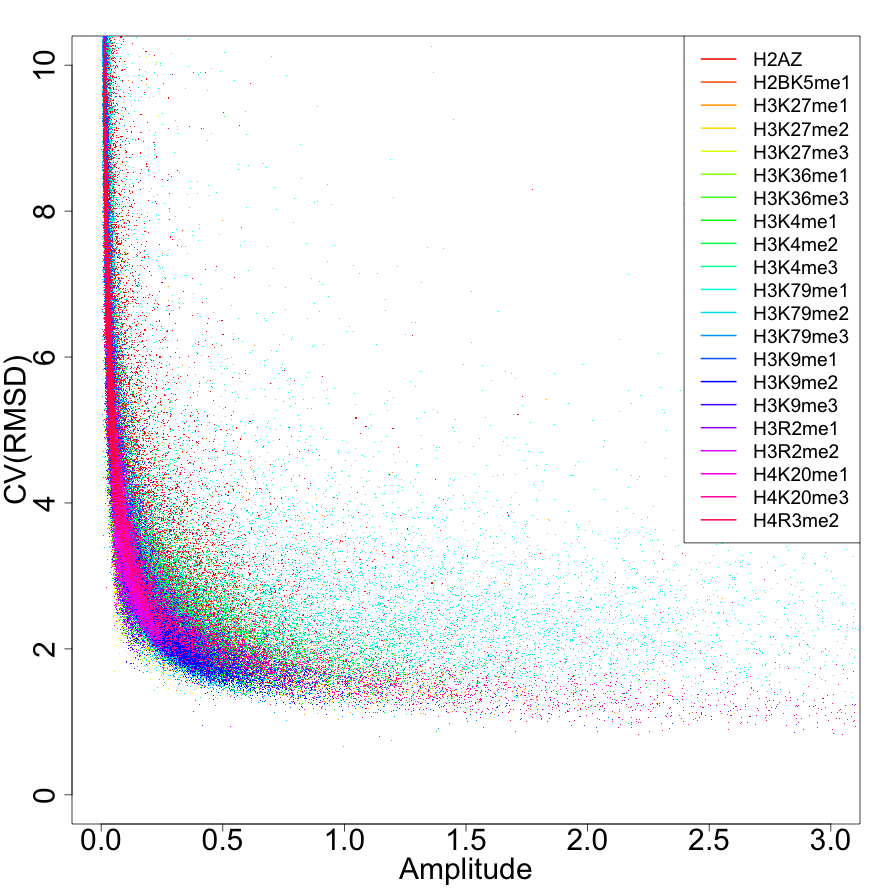

Supplement: Additional file 6 — Figure S3 Relative error of mark enrichment models. CV(RMSD) versus amplitude. Colors represent different marks as shown in the legend. Low amplitudes correspond to low levels/coverage, and thus high CV(RMSD) values. As amplitude increases, values reach an asymptotic value. [file 1471-2105-11-396-S6.PNG]

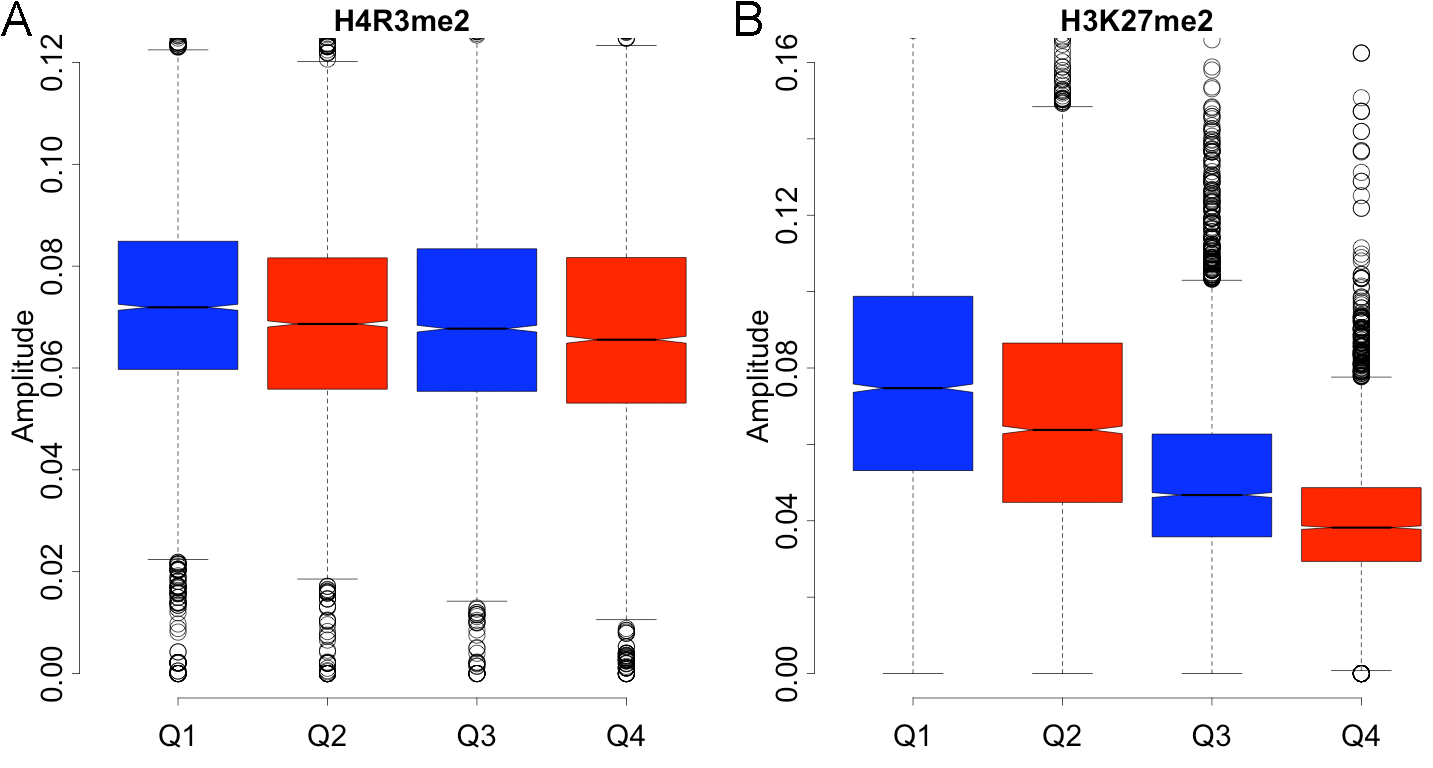

Supplement: Additional file 9 — Figure S4. Box plots of amplitudes across expression. Box plots of H4R3me2 (A) and H3K27me2 (B) amplitudes across the data stratified by quartiles of gene expression, where Q1 and Q4 represent the lowest and highest gene expression groups, respectively. [file 1471-2105-11-396-S9.PNG]

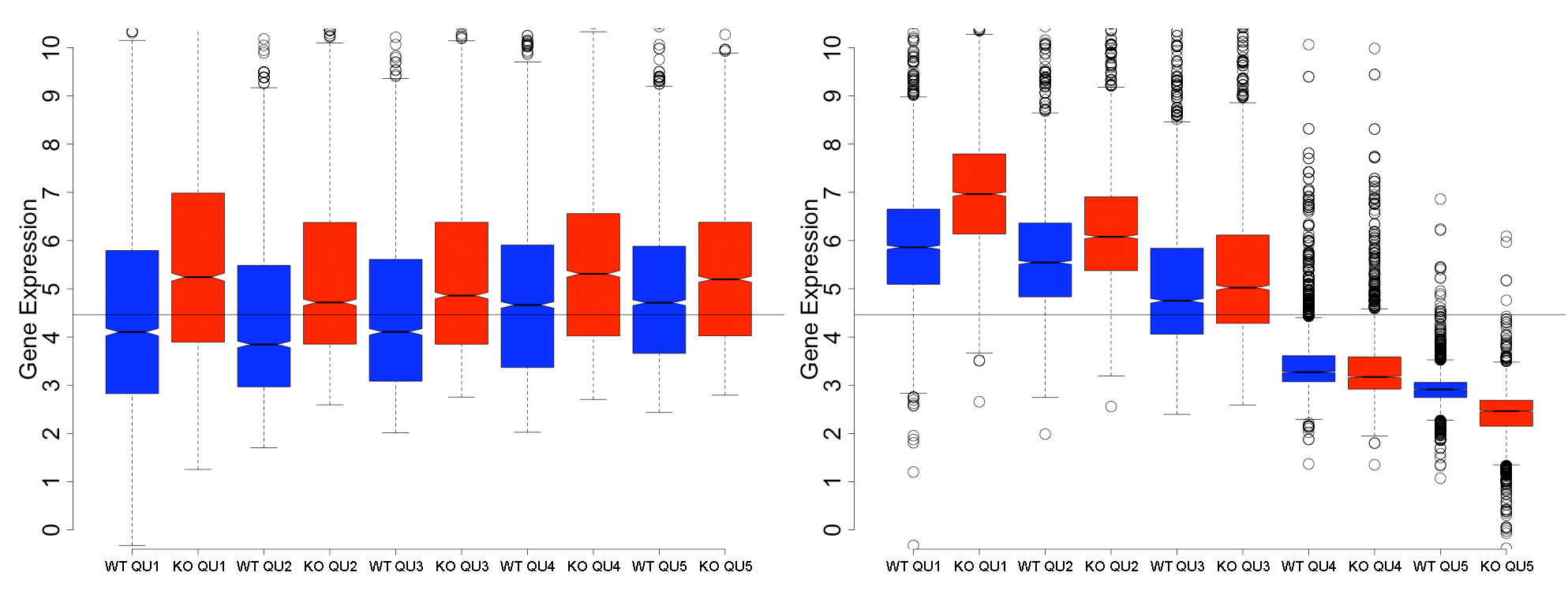

Supplement: Additional file 10 — Figure S5. Box plots of predicted gene expression before and after knockout. Box plots of predicted gene expression before and after knockout of (A) H4R3me2 and (B) H3K27me2. Plots are stratified along the x-axes by quintiles of log2 fold change (WT/KO) in gene expression predicted by the MLM. [file 1471-2105-11-396-S10.PNG]
